# Knowledge and Attitude of the General Population in Saudi Arabia Toward Weight Management Medications (WMMs): A Cross-Sectional Study

**DOI:** 10.7759/cureus.42875

**Published:** 2023-08-02

**Authors:** Malak A Algarni, Ameera Ali M Algarni, Waleed A Alqarni, Ahmad Y Alqassim

**Affiliations:** 1 Family Medicine, Postgraduate Program of Family Medicine, Ministry of Health, Jeddah, SAU; 2 Medicine, Ibn Sina National College for Medical Studies, Jeddah, SAU; 3 Clinical Nutrition, University of Nottingham, Nottingham, GBR; 4 Nutrition, Ministry of Education, Jeddah, SAU; 5 Family and Community Medicine, Faculty of Medicine, Jazan University, Jazan, SAU

**Keywords:** obesity management, weight loss, weight management drugs, saudi arabia, obesity intervention, public health interventions, pharmacological interventions, knowledge and attitudes, obesity, weight management medications (wmms)

## Abstract

Background: Over the past decades, the global prevalence of obesity has tripled, with the Kingdom of Saudi Arabia experiencing a notably higher rate of increase. While lifestyle modifications remain the first line of treatment, pharmacological interventions are often employed when dietary and exercise interventions prove insufficient. However, safety concerns, misuse, and limited knowledge about weight management medications (WMMs) pose serious challenges.

Objectives: The objectives of this study were to determine the level of knowledge and examine attitudes towards WMMs among the general population, and to explore the factors associated with these knowledge levels and attitude patterns.

Methods: A cross-sectional study was conducted among adults from the general population in Saudi Arabia from January 2023 to May 2023. Participants completed a validated, self-administered electronic questionnaire in Arabic language. The questionnaire captured sociodemographic, lifestyle and health data, knowledge about WMMs, and attitudes toward them. The outcome measures included knowledge and attitudes scores. Factors associated with knowledge and attitudes were analyzed using chi-square tests. Statistical significance was determined at a p-value of <0.05.

Results: Around 716 respondents were included in the final analysis. Most of the participants acknowledged diet 565 (78.9%) and exercise 621 (86.7%) as effective strategies to lose weight. Only 222 (31.0%) participants recognized pharmaceutical medications as a weight management strategy. Knowledge about specific weight loss medications varied, with the highest recognition for semaglutide (Ozempic®, Novo Nordisk, Bagsværd, Denmark) 236 (33.0%) and liraglutide (Saxenda®, Novo Nordisk, Bagsværd, Denmark) 228 (31.8%), while the other WMMs were not commonly known between participants. Regarding attitudes, the majority disagreed with statements that WMMs are more effective than diet/exercise 413 (57.7%), are safe 405 (56.6%), and are more convenient to use 408 (57.0%). Notably, about three-quarters [534 (74.6%)] of participants agreed that these medications require specialist’s counseling. No correlation was observed between knowledge score and attitude score (Pearson's correlation coefficient r=0.03; p=0.330). Respondents' knowledge about WMMs was significantly influenced by age, monthly income, educational level, psychiatric history, and previous use of WMMs (p<0.05). Adequate knowledge was more prevalent among participants aged 26-35, earning more than 20K SAR monthly, postgraduates, those with a psychiatric history, and past users of WMMs. Attitudes toward WMMs, however, showed no significant association with sociodemographic or health-related factors (p>0.05). However, prior use of WMMs significantly correlated with attitudes (p=0.007), with past users demonstrating more favorable attitudes.

Conclusion: This study reveals a limited knowledge and cautious attitude regarding WMMs in the Saudi population, despite the high prevalence of obesity. With prior use of WMMs correlating with better knowledge and more favorable attitudes, these findings emphasize the need for targeted interventions to enhance public awareness and safe usage of these medications.

## Introduction

Obesity has tripled over the past decades globally, with the Kingdom of Saudi Arabia (KSA) demonstrating a rate of increase that is higher than many other countries [1-2]. In 2002, the prevalence of obesity and overweight in Saudi Arabia was estimated at 37.8%, and it increased to 54.3% in 2018, representing approximately a 50% increase in the past two decades [3-4]. This is subsequent to the notable transition in Saudi's lifestyle from a traditional towards a more Western lifestyle [5]. Several recent studies have shown that the Saudi population shifted to a more sedentary lifestyle, with increased consumption of high-calorie food and drinks, while physical activity decreased [6-7]. Moreover, the COVID-19 pandemic changed the nutritional habits of Saudis. During the lockdown, 58.1% of Saudi participants in a study reported an increase in their food consumption, their fast food (47.1%), and sweets (48.5%) consumption, while 30.8% reported gaining weight throughout the lockdown [8].

Individuals with obesity already have an increased risk of developing the 21st century's most frequent non-communicable chronic diseases such as hypertension, cardiovascular disease, type 2 diabetes (T2D), and certain cancers [9]. Therefore, reducing the levels of obesity is considered a major public health goal in Saudi Arabia. The Ministry of Health considered the elaboration of evidence-based data to promote citizens’ health by targeting a number of chronic diseases and cardiovascular factors including obesity and overweight. In 2015, 12 guidelines have been established considering at first intention the lifestyle interventions and prioritizing individualized counseling interventions with supportive cognitive and behavioral interventions. On the other hand, due to a lack of solid evidence, no recommendations were emitted regarding specific types of diet over others [10]. Primary care physicians and dietitians play a key role in assisting individuals with creating effective dietary strategies to manage their obesity [8]. Nevertheless, previous research demonstrated low adherence to healthy diet guidelines among the Saudi population [11].

Pharmacological interventions are considered when diet and physical exercise are insufficient. According to Saudi guidelines 2022, pharmaceutical management of obesity that was recommended by a panel of experts includes the use of liraglutide and orlistat in obese and overweight adults [12]. Furthermore, bariatric surgery was approved for morbid obesity or type III obesity with comorbidities [10]. Metformin is commonly used off-label in Saudi Arabia as a weight-reducing agent. However, it is not US-FDA or SFDA-approved as a WMM.

However, tolerability and safety of anti-obesity medication remain an issue in long-term usage [13]. Indeed, during the last few years, due to substantial side effects, the majority of weight management medications (WMMs) that were approved and advertised were withdrawn [14]. Currently, the US FDA has approved orlistat, phentermine/topiramate, naltrexone/bupropion, and liraglutide as anti-obesity drugs, each with a specific mechanism of action [15]. Lorcaserin was approved by the US FDA in 2012 [16]; it suppresses appetite by stimulating 5-HT2C receptors on the pro-opiomelanocortin (POMC) neurons [17]. However, the US FDA announced the withdrawal of lorcaserin from the industry in February 2020, after a clinical trial that revealed a serious side effect of the drug: elevated risk of cancer [18].

Another alarming aspect of the issue that was observed in several countries is the increasing misuse of medications, specially WMMs, without medical indication or prescription [19-20]. Furthermore, the knowledge of community pharmacists, notably in Saudi Arabia, on the potential side effects of weight management products is inadequate [21]. Poor knowledge of community pharmacists impacts the public's awareness of the risks and adverse effects of drugs, thus influencing the public attitude and behavior toward these medications, especially considering the sources of medication that were reported to be family, friends, and other non-physicians [20, 22]. This highlights the safety and risks of misuse as a major public health concern, which emphasizes the need for data on public awareness and knowledge about the use and risks related to such drugs.

This study was conducted to assess knowledge and attitude toward WMMs among the public. We assessed the knowledge of the general population about the available WMMs on the market and explored their attitudes regarding the safe use of these pharmaceutical products for weight loss. Furthermore, we examined the associated sociodemographic, lifestyle, and clinical factors, and explored the correlation between knowledge levels and attitude patterns. By focusing on the general public, the research aims to identify gaps in awareness and perceptions, enabling targeted educational interventions to promote responsible usage. Such data are important in promoting the safe and effective use of these medications, guiding further research and public health interventions in this emerging area of health care.

## Materials and methods

Design and setting

A cross-sectional study was conducted, and participants were recruited from all regions of Saudi Arabia, between January and June 2023. The study protocol was ethically reviewed and approved by the Standing Committee for Scientific Research of Jazan University (Reference#: REC-44/09/599).

Population

Male and female adults aged 18 years and older, from the general population of Saudi Arabia were included. Individuals who use WMMs for diabetes mellitus were excluded. Participants with thyroid dysfunction and end-stage organ failure were also excluded. Participants below 18 years old and who declined to participate were excluded from the final dataset.

Sampling

The sample size was calculated to detect an unknown percentage (p=0.50) of adequate knowledge about WMMs, with a 95% confidence interval and 80% statistical power. A total of 377 individuals are necessary to meet the targeted statistical power. This goal was increased to N=400 to compensate for incomplete data. A convenience sampling was used to recruit all the consenting participants.

Tools

The study utilized a comprehensive, structured, and self-administered electronic questionnaire, which was designed by the primary investigator and subsequently validated by a family medicine consultant, a clinical nutrition specialist, and an epidemiologist. The questionnaire was administered in the Arabic language; however, the English version was used for ethical approval and statistical analysis. This questionnaire was segmented into four parts. Part A explored socio-demographic data such as age, gender, marital status, income, educational level, and health insurance status. Part B captured health and lifestyle data, subdivided into general health status (including history of eating disorders, other comorbidities, smoking, drug abuse, weight, height, and perception of own body image), dietary habits (previous consultation with a dietitian for weight management, prior diet, and perception of own dietary habits), and physical activity level. Part C assessed the participants' knowledge of weight management strategies, specifically their understanding of various weight management methods and their awareness of six WMMs: metformin (Glucophage®, Merck KGaA, Darmstadt, Germany), semaglutide (Ozempic, Wegovy®, Rybelsus®, Novo Nordisk, Bagsværd, Denmark), liraglutide (Saxenda), orlistat (Xenical®, Cheplapharm, Greifswald, Germany), phentermine/topiramate ER (Qysmia®, Vivus, Campbell, CA), and naloxone/bupropion (Contrave®, Orexigen Therapeutics, Inc., La Jolla, CA). Part D assessed attitudes toward WMMs, gathering respondents' level of agreement on statements such as "WMMs are more effective than diet and exercise," "WMMs are safe and pose no significant health risks," and "Weight loss achieved by using WMMs is long-lasting." The items were classified into two categories: "permissive" indicating a favorable stance, and "restrictive" for an unfavorable outlook on the use of WMMs; the latter category was scored using a reverse scale.

Pilot study

The questionnaire underwent pilot testing among 20 random participants, to test the validity and clarity of the items. The pilot sample of participants was not included in the main study.

Study outcomes

The present study comprises two dependent variables:

1) Knowledge about WMMs, indicated by a knowledge score (range 0-12) calculated as the number of weight-loss strategies (out of 6) and medications (out of 6) that were correctly identified by the participants. Adequate knowledge was determined as a knowledge score ≥7 out of 12.

2) Similarly, an attitude score (8-24) was calculated as the sum of the items’ scores and was dichotomized into unfavorable (score 8-15) and favorable (≥16).

Statistical methods

Data were analyzed using IBM Statistical Package for the Social Sciences (SPSS) for Windows, Version 21.0 (IBM Corp., Armonk, NY). Descriptive statistics were used to present the patterns of answers to the different questionnaire items. Cronbach’s alpha was calculated to evaluate the internal consistency of the study scales. Factors associated with knowledge (adequate vs inadequate) and attitudes (favorable vs unfavorable) were analyzed using chi-square test. A p-value <0.05 was used for statistical significance.

## Results

Sociodemographic data

The final analysis included 716 participants from all regions of Saudi Arabia regardless of nationality. It revealed that the majority were females 443 (61.9%), aged between 26 and 35 [261 (36.5%)], and of Saudi nationality 691 (96.5%). Most were married 384 (53.6%) and did not have children 402 (56.1%). Predominantly, participants were in the income bracket of less than 7K SAR 348 (48.6%), and their professional status was employed 338 (47.2%). The majority held a university degree 463 (64.7%) and did not have health insurance 494 (69.0%). Concerning residency, participants were approximately equally distributed among the center 207 (28.9%), east 187 (26.1%), and west 181 (25.3%) regions (Table 1).

**Table 1:** Sociodemographic data (N=716).

Parameter	Level	Frequency	Percentage
Gender	Male	273	38.1
	Female	443	61.9
Age	18-25	198	27.7
	26-35	261	36.5
	36-45	158	22.1
	46-50	51	7.1
	Above 50	48	6.7
Nationality	Saudi	691	96.5
	Non-Saudi	25	3.5
Marital status	Single	302	42.2
	Married	384	53.6
	Divorced	28	3.9
	Widowed	2	0.3
Number of children	None	402	56.1
	One	0	0.0
	Two or more	314	43.9
Monthly income	<7K SAR	348	48.6
	7K-15K SAR	219	30.6
	15K-20K SAR	96	13.4
	>20K SAR	53	7.4
Professional status	Housewife	157	21.9
	Employed	338	47.2
	Businessman/self employed	16	2.2
	Student	183	25.6
	Retired	22	3.1
Educational level	Primary or lower	5	0.7
	Middle school	14	2.0
	Secondary school	142	19.8
	University	463	64.7
	Post-graduate	92	12.8
Health insurance status	None	494	69.0
	Through employer	199	27.8
	Purchased directly	23	3.2
Residency region	North	75	10.5
	South	66	9.2
	Center	207	28.9
	East	187	26.1
	West	181	25.3

Health and lifestyle data

Analysis of health and lifestyle data for the 716 participants revealed that dyslipidemia 166 (23.2%) and eating disorders 124 (17.3%) were the most common chronic diseases among participants. The majority were non-smokers 586 (81.8%) and fell within the normal weight 281 (39.2%) or overweight 263 (36.7%) categories. About 280 (39.1%) had considered and succeeded in a weight-loss program, while 196 (27.4%) attempted but failed. Most participants never consulted a dietitian 496 (69.3%) or a coach 487 (68.0%). Slightly more than half had never or only once followed a diet to lose weight [266 (37.2%) and 148 (20.7%) respectively] and never or only once followed a workout program [303 (42.3%) and 153 (21.4%) respectively]. The majority were somewhat watchful of their eating habits 415 (58.0%). Participants predominantly had a low-activity job 282 (39.4%) and exercised almost never or one to two times per week [308 (43.0%) and 233 (32.5%) respectively]. Approximately 256 (35.8%) reported walking durations of less than 30 min (Table 2).

**Table 2:** Health and lifestyle data (N=716).

Parameter	Level	Frequency	Percentage
Chronic diseases	Hypertension	43	6.0
	Type 2 diabetes	33	4.6
	Thyroid dysfunction	44	6.1
	Dyslipidemia	166	23.2
	Eating disorder	124	17.3
	Asthma	12	1.7
	GIT (Ulcer, IBS…etc.)	11	1.5
	Psychiatric history	48	6.7
	Other chronic disease	63	8.8
No. comorbidities	None	398	55.6
	1	179	25.0
	2	80	11.2
	3+	59	8.2
Smoking	None-smoker	586	81.8
	Ex-smoker	28	3.9
	Current smoker	102	14.2
Weight-range	Underweight	54	7.5
	Normal weight	281	39.2
	Overweight	263	36.7
	Obese	118	16.5
Weight loss consideration	Never	143	20.0
	Yes, but never attempted	97	13.5
	Yes, but failed	196	27.4
	Yes, and succeeded	280	39.1
Dietitian consult	Never	496	69.3
	Only once	128	17.9
	More than once	92	12.8
Diet to lose weight	Never	266	37.2
	Only once	148	20.7
	More than once	302	42.7
Eating habits	Not watchful	264	36.9
	Somewhat watchful	415	58.0
	Very watchful	37	5.2
Physical exercise rhythm	Almost never	308	43.0
	1-2 per week	233	32.5
	3-4 per week	132	18.4
	5 or more per week	43	6.0
Walking duration	None	182	25.4
	<30 min	256	35.8
	30-60 min	218	30.4
	>1 h	60	8.4
Job type	Sedentary	255	35.6
	Low activity	282	39.4
	Moderate activity	156	21.8
	High activity	23	3.2
Coach consult	Never	487	68.0
	Only once	135	18.9
	More than once	94	13.1
Workout program	Never	303	42.3
	Only once	153	21.4
	More than once	260	36.3

Knowledge about weight management strategies

The knowledge of participants about weight management strategies was limited. The majority acknowledged diet 565 (78.9%) and exercise 621 (86.7%) as effective strategies but had less consensus on the use of herbal products 148 (20.7%), pharmaceutical drugs 222 (31.0%), cognitive and behavioral therapy 333 (46.5%), and surgery 325 (45.4%). Knowledge about specific weight-loss medications varied, with the highest recognition for semaglutide (Ozempic, Wegovy®, Rybelsus®) 236 (33.0%) and liraglutide (Saxenda) 228 (31.8%), while naloxone/bupropion (Contrave®) and phentermine/topiramate ER (Qysmia®) had the least recognition [43 (6.0%) and 42 (5.9%) respectively]. Uncertainty was particularly high for phentermine/topiramate ER (Qysmia®), naloxone/bupropion (Contrave), and orlistat (Xenical®, Cheplapharm, Greifswald, Germany) with 422 (58.9%), 416 (58.1%), and 384 (53.6%) respectively answered "Not sure" if used for weight loss (Table 3).

**Table 3:** Knowledge about weight management strategies (N=716).

Question	Item	No (%)	Yes (%)	Not sure (%)
Among the following items, which are strategies that can be used to lose weight?	Diet	97 (13.5)	565 (78.9)	54 (7.5)
	Exercise	60 (8.4)	621 (86.7)	35 (4.9)
	Herbal products	411 (57.4)	148 (20.7)	157 (21.9)
	Pharmaceutical drugs	375 (52.4)	222 (31.0)	119 (16.6)
	Cognitive and behavioral therapy	236 (33.0)	333 (46.5)	147 (20.5)
	Surgery	330 (46.1)	325 (45.4)	61 (8.5)
Among the following drugs, which can be used to lose weight?	Liraglutide (Saxenda®)	184 (25.7)	228 (31.8)	304 (42.5)
	Metformin (Glucophage®)	252 (35.2)	124 (17.3)	340 (47.5)
	Orlistat (Xenical®)	209 (29.2)	123 (17.2)	384 (53.6)
	Semaglutide (Ozempic®, Wegovy®, Rybelsus®)	186 (26.0)	236 (33.0)	294 (41.1)
	Naloxone/bupropion (Contrava®)	257 (35.9)	43 (6.0)	416 (58.1)
	Phentermine/topiramate ER (Qysmia®)	252 (35.2)	42 (5.9)	422 (58.9)

Attitudes toward WMMs

Participants demonstrated cautious attitudes toward WMMs. The majority disagreed with statements that WMMs are more effective than diet/exercise 413 (57.7%), are safe 405 (56.6%), and are more convenient to use 408 (57.0%), resulting in low favorability scores of 1.49, 1.48, and 1.60 respectively. Participants were split between disagree 166 (23.2%) and agree 165 (23.0%) on whether WMMs enable rapid results, with a favorability score of 2.00. Similarly, for the statement concerning the long-lasting effect of WMMs on weight loss, most answers were neutral 382 (53.4%) or disagreed 221 (30.9%), yielding a favorability score of 1.85. Restrictive attitudes also varied, with the majority agreeing that WMMs require specialist's counseling 534 (74.6%) and should be restricted to those who failed to lose weight with diet/exercise 303 (42.3%). Most participants agreed that WMMs should only be used by people with extreme obesity 367 (51.3%). Favorability scores were 1.34, 1.78, and 1.65 respectively for these statements (Table 4).

**Table 4:** Attitudes toward WMMs. WMMs, weight management medications; SD, standard deviation *Reverse-scoring item (favorability score takes into consideration the type of the attitude and expresses how favorable is the participant’s answer to WMMs in the given item).

Attitude	Type	Disagree	Neutral	Agree	Favorability score (mean, SD: out of 3)
WMMs are more effective than diet/exercise	Permissive	413 (57.7)	252 (35.2)	51 (7.1)	1.49 (0.63)
WMMs should be restricted to those who failed to lose weight with diet/exercise	Restrictive *	142 (19.8)	271 (37.8)	303 (42.3)	1.78 (0.76)
WMMs are safe	Permissive	405 (56.6)	281 (39.2)	30 (4.2)	1.48 (0.58)
WMMs require a specialist’s counseling	Restrictive	58 (8.1)	124 (17.3)	534 (74.6)	1.34 (0.62)
WMMs are more convenient to use	Permissive	408 (57.0)	183 (25.6)	125 (17.5)	1.60 (0.77)
WMMs enable rapid results	Permissive	166 (23.2)	385 (53.8)	165 (23.0)	2.00 (0.68)
Weight loss with WMMs is long-lasting	Permissive	221 (30.9)	382 (53.4)	113 (15.8)	1.85 (0.67)
WMMs should only be for people with extreme obesity	Restrictive	117 (16.3)	232 (32.4)	367 (51.3)	1.65 (0.74)

Reliability of the study scales

Reliability analysis showed the scale for knowledge about weight management strategies was highly reliable (Cronbach's alpha = 0.885) with a mean score of 4.20, standard deviation (SD) of 2.50, and range of 0-12. The attitudes toward WMMs scale exhibited modest reliability (Cronbach's alpha = 0.675) with a mean score of 13.18, SD of 2.30, and range of 8-21. Higher scores indicate better knowledge and more favorable attitudes (Table 5). Notably, no correlation was observed between knowledge score and attitude score (Pearson's correlation coefficient r = 0.03; p=0.330).

**Table 5:** Reliability analysis of the study scales. Depending on the scale, higher scores indicate higher knowledge and more favorable attitudes.

Scale	No. items	Cronbach’s alpha	Score statistics		
			Mean	SD	Range
Knowledge about weight management strategies	12	0.885	4.20	2.50	0-12
Attitudes toward WMMs	8	0.675	13.18	2.30	8-21

Factors associated with knowledge about WMMs

Table 6 presents the factors associated with knowledge about weight management. Age was found to be a key influencer, as those between 26 and 35 years old were most likely to have adequate knowledge (p=0.010*). Monthly income also showed to be a significant factor, with respondents earning more than 20K SAR displaying the highest level of adequate knowledge (p<0.001*). Education was another significant factor, as postgraduates demonstrated the most extensive knowledge (p=0.020*). Remarkably, respondents with a psychiatric history showed considerably higher knowledge levels (p=0.005*), and those who had used WMMs in the past were significantly more likely to have adequate knowledge than those who had not (p<0.001*). These results highlighted the influence of socioeconomic factors, health conditions, and previous use on knowledge levels about WMMs.

**Table 6:** Factors associated with knowledge. WMM, weight management medication; GIT, gastrointestinal tract; IBS, irritable bowel syndrome

Factor	Level	Inadequate level		Adequate		p-Value
		N	%	N	%	
Gender	Male	203	74.4	70	25.6	
	Female	310	70.0	133	30.0	0.206
Age	18-25	141	71.2	57	28.8	
	26-35	169	64.8	92	35.2	
	36-45	124	78.5	34	21.5	
	46-50	42	82.4	9	17.6	
	Above 50	37	77.1	11	22.9	0.010*
Nationality	Saudi	493	71.3	198	28.7	
	Non-Saudi	20	80.0	5	20.0	0.346
Marital status	Single	213	70.5	89	29.5	
	Married	278	72.4	106	27.6	
	Divorced	20	71.4	8	28.6	
	Widowed	2	100.0	0	0.0	0.781
Children	No	278	69.2	124	30.8	
	Yes	235	74.8	79	25.2	.094
Monthly income	<7K SAR	274	78.7	74	21.3	
	7K-15K SAR	149	68.0	70	32.0	
	15K-20K SAR	62	64.6	34	35.4	
	>20K SAR	28	52.8	25	47.2	<0.001*
Professional status	Housewife	123	78.3	34	21.7	
	Employed	230	68.0	108	32.0	
	Businessman/self employed	13	81.3	3	18.8	
	Student	128	69.9	55	30.1	
	Retired	19	86.4	3	13.6	0.062
Educational level	Primary or lower	4	80.0	1	20.0	
	Middle school	10	71.4	4	28.6	
	Secondary school	115	81.0	27	19.0	
	University	328	70.8	135	29.2	
	Post-graduate	56	60.9	36	39.1	0.020*
Health insurance status	None	345	69.8	149	30.2	
	Through employer	152	76.4	47	23.6	
	Purchased directly	16	69.6	7	30.4	0.219
Residency region	North	53	70.7	22	29.3	
	South	48	72.7	18	27.3	
	Center	162	78.3	45	21.7	
	East	131	70.1	56	29.9	
	West	119	65.7	62	34.3	0.097
Hypertension	No	485	72.1	188	27.9	
	Yes	28	65.1	15	34.9	0.327
Type 2 diabetes	No	493	72.2	190	27.8	
	Yes	20	60.6	13	39.4	0.150
Thyroid dysfunction	No	482	71.7	190	28.3	
	Yes	31	70.5	13	29.5	0.856
Dyslipidemia	No	396	72.0	154	28.0	
	Yes	117	70.5	49	29.5	0.704
Eating disorder	No	426	72.0	166	28.0	
	Yes	87	70.2	37	29.8	0.686
Asthma	No	506	71.9	198	28.1	
	Yes	7	58.3	5	41.7	0.302
GIT (ulcer, IBS..)	No	503	71.3	202	28.7	
	Yes	10	90.9	1	9.1	0.153
Psychiatric history	No	487	72.9	181	27.1	
	Yes	26	54.2	22	45.8	0.005*
Other chronic disease	No	467	71.5	186	28.5	
	Yes	46	73.0	17	27.0	0.801
Number of comorbidities	None	294	73.9	104	26.1	
	1	129	72.1	50	27.9	
	2	49	61.3	31	38.8	
	3+	41	69.5	18	30.5	0.146
Use of WMMs	Never	477	75.4	156	24.6	
	Ever	36	43.4	47	56.6	<0.001>

Factors associated with attitudes toward WMMs

Table 7 examines factors associated with attitudes toward WMMs. Notably, none of the sociodemographic or health-related factors showed a statistically significant association with attitudes toward WMMs (p>0.05). However, the use of WMMs did show a significant correlation (p=0.007*), with those who have ever used WMMs exhibiting a more favorable attitude than those who have never used them. It is worth mentioning that females were found to be slightly more likely to have an unfavorable attitude toward WMMs compared to males (84.7% vs 79.9%). Attitudes seemed to be slightly less favorable among those with post-graduate education (90.2% unfavorable) compared to university degree holders (81.9% unfavorable). Participants with an eating disorder had a higher percentage of unfavorable attitudes (87.1%) compared to those without (81.9%). These nuances, while not statistically significant, may still be important to consider in a comprehensive understanding of attitudes toward WMMs.

**Table 7:** Factors associated with attitude. WMM, weight management medication; GIT, gastrointestinal tract; IBS, irritable bowel syndrome

Factor	Level	Unfavorable		Favorable		p-Value
		N	%	N	%	
Gender	Male	218	79.9	55	20.1	
	Female	375	84.7	68	15.3	0.098
Age	18-25	157	79.3	41	20.7	
	26-35	223	85.4	38	14.6	
	36-45	133	84.2	25	15.8	
	46-50	41	80.4	10	19.6	
	Above 50	39	81.3	9	18.8	0.479
Nationality	Saudi	572	82.8	119	17.2	
	Non-Saudi	21	84.0	4	16.0	0.874
Marital status	Single	247	81.8	55	18.2	
	Married	319	83.1	65	16.9	
	Divorced	25	89.3	3	10.7	
	Widowed	2	100.0	0	0.0	0.687
Children	No	327	81.3	75	18.7	
	Yes	266	84.7	48	15.3	0.236
Monthly income	<7K SAR	286	82.2	62	17.8	
	7K-15K SAR	180	82.2	39	17.8	
	15K-20K SAR	82	85.4	14	14.6	
	>20K SAR	45	84.9	8	15.1	.855
Professional status	Housewife	134	85.4	23	14.6	
	Employed	284	84.0	54	16.0	
	Businessman/self employed	13	81.3	3	18.8	
	Student	145	79.2	38	20.8	
	Retired	17	77.3	5	22.7	0.524
Educational level	Primary or lower	3	60.0	2	40.0	
	Middle school	9	64.3	5	35.7	
	Secondary school	119	83.8	23	16.2	
	University	379	81.9	84	18.1	
	Post-graduate	83	90.2	9	9.8	0.058
Health insurance status	None	404	81.8	90	18.2	
	Through employer	171	85.9	28	14.1	
	Purchased directly	18	78.3	5	21.7	0.356
Residency region	North	62	82.7	13	17.3	
	South	54	81.8	12	18.2	
	Center	173	83.6	34	16.4	
	East	157	84.0	30	16.0	
	West	147	81.2	34	18.8	0.960
Hypertension	No	557	82.8	116	17.2	
	Yes	36	83.7	7	16.3	0.872
Type 2 diabetes	No	565	82.7	118	17.3	
	Yes	28	84.8	5	15.2	0.752
Thyroid dysfunction	No	554	82.4	118	17.6	
	Yes	39	88.6	5	11.4	0.291
Dyslipidemia	No	453	82.4	97	17.6	
	Yes	140	84.3	26	15.7	0.555
Eating disorder	No	485	81.9	107	18.1	
	Yes	108	87.1	16	12.9	0.165
Asthma	No	584	83.0	120	17.0	
	Yes	9	75.0	3	25.0	0.469
GIT (ulcer, IBS, etc.)	No	584	82.8	121	17.2	
	Yes	9	81.8	2	18.2	0.929
Psychiatric history	No	553	82.8	115	17.2	
	Yes	40	83.3	8	16.7	0.922
Other chronic disease	No	542	83.0	111	17.0	
	Yes	51	81.0	12	19.0	0.680
Number of comorbidities	None	327	82.2	71	17.8	
	1	146	81.6	33	18.4	
	2	70	87.5	10	12.5	
	3+	50	84.7	9	15.3	0.636
Use of WMMs	Never	533	84.2	100	15.8	
	Ever	60	72.3	23	27.7	0.007*

## Discussion

Summary of findings

In light of the escalating obesity epidemic in the Kingdom of Saudi Arabia, worsened by lifestyle changes and the recent COVID-19 pandemic, we conducted this study to assess public understanding and attitudes toward FDA-approved and other commonly used WMMs. Our focus on knowledge and attitudes, alongside the exploration of related socio-demographic, lifestyle, and clinical factors, provides a comprehensive overview of this growing public health concern.

Our study sample encompassed 716 participants, predominantly females 443 (61.9%) aged 26-35 years 261 (36.5%), with a majority holding a university degree 463 (64.7%) and situated in the middle to lower income brackets [219 (30.6%) and 348 (48.6%) respectively]. Their health profiles showed dyslipidemia and eating disorders as the most common chronic conditions, with a majority falling within the normal weight or overweight categories. Weight management strategies were recognized, with diet and exercise being acknowledged as effective by the majority, while there was less consensus on the use of herbal products, pharmaceutical drugs, cognitive and behavioral therapy, and surgery. Knowledge was limited about specific WMMs. Participants generally demonstrated a cautious attitude toward WMMs, with low favorability scores for the statements asserting their effectiveness, safety, and convenience. Nevertheless, it is important to consider these findings in the broader context of understanding knowledge and attitudes related to weight management. Age, income, education, psychiatric history, and past usage of WMMs emerged as significant factors influencing knowledge about weight management. Interestingly, none of the sociodemographic or health-related factors exhibited a statistically significant association with attitudes toward WMMs, except for prior use of these drugs. This study provides crucial insights into the complex interplay of personal, socio-economic, and health factors influencing public knowledge and attitudes concerning weight management strategies.

Knowledge about WMMs

The present study showed variable levels of knowledge about weight management strategies. While diet and exercise were largely acknowledged as effective strategies, pharmaceutical drugs, cognitive and behavioral therapy, and surgery were only recognized by a minority of the participants. Additionally, knowledge about specific WMMs was generally low. The most recognized drugs, i.e. liraglutide (Saxenda) and semaglutide (Ozempic, Wegovy®, Rybelsus®), were reported by about two-thirds of the participants.

Only a few studies have investigated the Saudi’s knowledge and attitude toward WMMs. A 2020 cross-sectional study by Hasan and Ganesh explored the levels of use of orlistat and metformin among 820 female participants. Results showed that 21.3% of the participants used either drug for weight loss, while more than 60% used diet and physical exercise for the same purpose. Of the two medications, orlistat was the most frequently used. The pharmacological method of weight loss was found to be positively correlated to women’s age and marital status. In fact, married Saudi women took more weight loss pills than others. Females between 19 and 25 of age consumed anti-obesity drugs the most [23]. On the other hand, there is more literature available about healthcare professionals’ knowledge and practice in anti-obesity drugs. In 2014, Al-Shammari provided evidence for the under-prescription of anti-obesity drugs in Eastern Saudi Arabia. In this cross-sectional study, 72.3% of physicians said they had never used weight loss medications for the management of obesity. Furthermore, even when required, WMMs were underutilized [24]. Al-Khaldi et al. reported that only 10% of practitioners were knowledgeable of the indications to administer WMMs [25]. Other data suggest that physicians were notified about the unavailability of anti-obesity medications in primary care in 2020 [26].

The findings of this study and the review of previous research highlight significant unfavorability in both the public and professionals toward WMMs use. A key observation is the low recognition and utilization of these drugs, with a clear preference for dietary and physical exercise strategies. The limited use of WMMs in practice, coupled with the prevalent low favorability, calls for more comprehensive education on these interventions. For the public, the focus should be on providing accurate information about the variety of weight management strategies available, including the role and effectiveness of specific medications. For healthcare professionals, the challenge is to promote a better understanding of the indications and usage of these medications. This is of utmost importance given that available literature suggests a degree of under-prescription and under-utilization of WMMs in primary care.

Attitudes towards WMMs

Overall, participants’ attitudes toward WMMs were cautious, demoting misunderstanding or skepticism toward their effectiveness, safety, and convenience. Such uncertainty could lead to lower compliance or outright rejection of these drugs, even when prescribed by healthcare professionals. This could ultimately hinder the successful management of weight-related health issues. Therefore, it is vital to address these misconceptions and enhance patient education about these medications to improve their acceptance and utilization in a clinical setting. Unexpectedly, we observed no correlation of attitudes with knowledge.

Data regarding patients’ attitudes toward WMMs is scarce. However, several studies explored the attitude of prescribers and other healthcare professionals. For instance, Foster et al. study in 2003 explored US physicians' attitudes toward obese patients, as well as their perceptions about the causes and treatment of obesity. Of the 620 respondents from a broad national sample, the consensus was that physical inactivity was recognized as the leading cause of obesity, with other behavioral factors such as overeating and high-fat diets. Regrettably, over half of the physicians held negative stereotypes about obese patients, characterizing them as awkward, unattractive, ugly, and noncompliant. When it came to treatment, obesity intervention was deemed less effective than therapies for most other chronic conditions. However, physicians were realistic about weight loss outcomes, agreeing that a 10% reduction in weight can significantly improve obesity-related health complications. Moreover, most of the respondents were willing to dedicate more time to weight management if they received appropriate compensation [27].

The observed attitudes and perceptions toward obese patients and weight management strategies present significant challenges for effective obesity management. Negative stereotypes held by physicians could potentially impact the patient-doctor relationship and hamper effective communication, leading to suboptimal care. Consequently, addressing these perceptions and attitudes among healthcare professionals is crucial to improve the management of obesity.

On the other hand, despite recognizing obesity as a medically significant issue, a study of French general practitioners revealed a lack of confidence and satisfaction in obesity management, with only 42% considering themselves well-prepared and 51% finding it professionally rewarding. Their unrealistic weight loss goals and low reliance on collaboration with dietitians further complicate obesity management. Moreover, the majority doubted the effectiveness of available drugs. This indicates a need for improved medical education, better collaboration with dietitians, and realistic goal-setting to enhance practices in managing obesity [28].

In Bahrain, a study by Al-Ghawi and Uauy revealed that while physicians acknowledge the severity of the obesity epidemic and express a willingness to take up a significant role in obesity management, their practices and attitudes are hindered by several barriers. Despite their skepticism toward the success rates of weight management, 60% of physicians feel capable of playing a major part in tackling obesity. Their knowledge about weight-loss goals is commendable, and they commonly incorporate obesity identification in chronic disease care. Nevertheless, only 36% of the respondents agree that their weight-management practices are effective. In terms of weight-loss strategies, physicians in Bahrain reportedly utilize a broad range of strategies, excluding pharmacotherapy and surgery. The primary challenges affecting patient care, as identified by the physicians, include time constraints, the absence of specialty clinics, a lack of clear guidelines, and an inadequate number of dietitians. The study concludes that the potential for improved obesity management practices is promising in Bahrain, provided the physicians receive adequate training and the identified obstacles in their working environment are effectively addressed [29].

The studies collectively highlight that attitudes toward obesity and WMMs among both patients and healthcare professionals significantly influence the approach to and efficacy of obesity treatment. Patients' skepticism about WMMs and their safety could hinder the successful management of obesity, underscoring the importance of robust patient education and dispelling misconceptions. However, the attitudes of healthcare professionals are equally critical. Negative stereotypes held by physicians about obese patients can lead to suboptimal care, emphasizing the need to address these perceptions. Across different countries, the confidence and satisfaction of healthcare professionals in managing obesity vary, often hindered by barriers such as time constraints, inadequate support, and unrealistic expectations. These observations suggest that comprehensive strategies involving patient education, improved medical training, and systemic changes in healthcare settings are needed to optimize obesity management practices. These observations underscore a global need for improved training, realistic goal-setting, and better inter-professional collaboration in obesity management. Furthermore, these findings should guide future research and policy initiatives to enhance obesity management on a global scale.

Sociodemographic and health factors

The observed associations highlight that knowledge about WMMs is influenced by various socioeconomic and health-related factors. Notably, younger age (26-35 years), higher income, higher education level, a psychiatric history, and prior use of WMMs are associated with better knowledge about these medications. This suggests that a certain level of exposure and experience, coupled with socioeconomic advantages, might facilitate better understanding and informed perspectives about WMMs.

However, it is intriguing to see that attitudes toward WMMs neither demonstrate a significant association with these same factors, nor with knowledge level. While knowledge is evidently influenced by sociodemographic and health-related conditions, attitudes seem to be more dependent on personal experience with the drugs. This could indicate that attitudes are shaped more by personal encounters and subjective experiences than by knowledge per se.

It is noteworthy that despite the lack of statistical significance, there are certain trends in attitudes worth considering. Females, postgraduates, and those with eating disorders tend to have slightly more unfavorable attitudes toward WMMs. Although these differences are not statistically significant, they might hint at underlying disparities that could become more apparent in larger or more specific population samples.

In essence, while knowledge about WMMs might be improved by elevating socioeconomic status, enhancing education, and increasing exposure to drugs, shifting attitudes may require more personalized interventions. Attitudes could be influenced by experiences, beliefs, cultural norms, or personal health conditions, and thus might not change solely with increased knowledge. This underscores the complexity of managing obesity and the need for holistic, personalized approaches that take into account not just the patient's knowledge, but also their attitudes and experiences.

Limitations

The study’s main limitation stems from its cross-sectional design. The use of a self-administered questionnaire could lead to response bias due to misunderstandings or social desirability influence. While the study investigated various sociodemographic, lifestyle, and clinical factors, other potential determinants of knowledge and attitudes toward WMMs, such as healthcare literacy and access to it, may have been overlooked. Finally, as the study was conducted in Saudi Arabia, cultural factors may limit the generalizability of results to other cultural contexts.

## Conclusions

This study provides valuable insights into the understanding and attitudes toward FDA-approved and other commonly used WMMs among the general population in Saudi Arabia. It reveals significant variations and limited knowledge about weight management strategies, specifically WMMs. Furthermore, it highlights the general uncertainty and lack of consensus regarding the effectiveness, safety, and ease of use of these pharmaceutical products. Socioeconomic factors, such as age, income, and education level, as well as psychiatric history and past use of WMMs, have been found to significantly influence knowledge levels. However, these factors, with the exception of prior WMM use, do not significantly affect attitudes toward these drugs.

These findings might shed light on the need for targeted educational initiatives and public health interventions aimed at increasing public knowledge and guiding attitudes about WMMs. As obesity continues to be a global health concern, it is crucial to promote the safe and effective use of these medications while mitigating their potential misuse. The information gathered in this study may serve as a foundational basis for future research and health interventions, ultimately contributing to more informed and effective weight management strategies. Future research could focus on the practices of the Saudi population when using WMMs, their reasons and motivators to use them, and their understanding of potential side effects.
